# Network pharmacological mechanism analysis and evidence-based medical validation of Dahuang Mudan Decoction in the treatment of acute pancreatitis

**DOI:** 10.1097/MD.0000000000039679

**Published:** 2024-09-13

**Authors:** Jinhan Chen, Mengjie Jiang, Yuou Ying, Yuan Ji, Yuying Chi, Linghui Tao, Fuping Wu, Mingxian Chen

**Affiliations:** a The Second Clinical Medical College of Zhejiang Chinese Medical University, Hangzhou, China; b Hangzhou TCM Hospital Affiliated to Zhejiang Chinese Medical University, Hangzhou, China; c School of Basic Medical Sciences, Zhejiang Chinese Medical University, Hangzhou, China; d Department of Gastroenterology, Tongde Hospital of Zhejiang Province, Hangzhou, China.

**Keywords:** acute pancreatitis, Dahuang Mudan Decoction, evidence-based medicine, meta-analysis, network pharmacology

## Abstract

**Background:**

Dahuang Mudan Decoction is commonly used in China for the treatment of acute pancreatitis. Nevertheless, the therapeutic efficacy of the drug remains a subject of debate, and its active ingredients and potential therapeutic targets remain to be determined. The present study used a network pharmacological approach to investigate the active ingredients and possible targets of the drug, and illustrated the clinical effectiveness of Dahuang Mudan Decoction in the treatment of acute pancreatitis by meta-analysis.

**Methods:**

The present study investigated the active ingredients of the constituent herbs of Dahuang Mudan Decoction using the TCMID database. In order to further identify molecular targets, Swiss Target Prediction, OMIM and Genecards databases was be used. The present study used metascape database for gene ontology function enrichment analysis and Kyoto Genome Encyclopedia pathway enrichment analysis. A gene interaction network diagram was established for predicting the main targets and mechanism of action to Dahuang Mudan Decoction for acute pancreatitis. To further illustrate the validity of the gene targets and the clinical efficacy of the drug, 13 relevant studies were included for meta-analysis and analyzed using the Cochrane Collaboration’s Review Manager 5.4 software.

**Result:**

After a thorough screening process, the present study identified three main components of Dahuang Mudan Decoction: kaempferol, quercetin and eupatin. These three major components have the potential to target 5 important proteins: AKT1, TNF-a, IL-6, TP53, HIF1A. In addition, pathway analyses by the Kyoto Genome Encyclopedia showed that Dahuang Mudan Decoction is active through the Pathways in cancer, AGE-RAGE signaling pathway in diabetic complications, PI3K-Akt signaling pathway, etc signaling pathway to act on acute pancreatitis. The results of meta-analysis showed that compared with the control group, the experimental group had superior performance in terms of overall treatment efficacy, reduction of hospital stays and inflammatory factor levels after treatment.

**Conclusion:**

In summary, network pharmacological studies have shown that Dahuang Mudan Decoction affects acute pancreatitis through different components, targets, and mechanisms. In addition, the meta-analysis study strongly supported the effectiveness of Dahuang Mudan Decoction in the treatment of acute pancreatitis.

## 1. Introduction

Acute pancreatitis develops due to the abnormal activation of pancreatic enzymes in the pancreas, leading to digestive effects in the gland itself and surrounding tissues and organs. It is characterized by a localized inflammatory response in the pancreas as the primary feature, and in severe cases, it leads to organ dysfunction and clinical manifestations of acute abdomen.^[1]^ In most mild cases, clinical remission is efficient with active treatment such as appropriate fluid resuscitation, pain and nausea control, etc. When acute pancreatitis develops into severe pancreatitis or progresses into chronic pancreatitis, it seriously jeopardizes the life and health of the patient. According to the relevant research department, the incidence of this disease is ranked as the second most common digestive disease, second only to appendicitis, and the global incidence of acute pancreatitis is increasing year by year.^[2,3]^

Dahuang Mudan Decoction is from “The Essentials of the Golden Chamber” by Zhang Zhongjing of the Eastern Han Dynasty. The formula includes Dahuang, which clears fire and expels blood stasis, acts as a laxative, and detoxifies. Mudan Pi cools blood and clears heat, promotes blood circulation, and dissipates blood stasis. The combination is effective for diarrhea caused by damp-heat stagnation in the intestines and bowels. Mangxiao disperses solid masses, aiding Dahuang in clearing solid heat. Taoren can activate blood and resolve stasis. Donggua Ren can induce diuresis to drain dampness, drains pus and eliminates carbuncles. The whole formula helps clear the intestines, eliminate carbuncles, and thus stops the pain.^[4]^

Dahuang Mudan Decoction is commonly used in clinical practice to treat abdominal symptoms, inflammatory aggregation, and patient prognosis caused by acute pancreatitis.^[5]^ A large number of clinical and experimental studies have proven that Dahuang Mudan Decoction has good anti-inflammatory, decongestive, immune-regulating, and blood circulation-improving effects. However, the molecular mechanism of Dahuang Mudan Decoction in the treatment of acute pancreatitis is still unclear, which limits its promotion and development.^[6]^

Network pharmacology is a valuable tool for analyzing the complex relationships between drug components, protein targets, diseases and genes. This method has been widely used in the analysis of Chinese medicinal preparations. The present study used network pharmacology to select the active ingredients of Dahuang Mudan Decoction, identify its targets, and analyze the genes involved in the treatment of acute pancreatitis. The aim of the study was to investigate the potential links between the active ingredients of Dahuang Mudan Decoction and the targets of acute pancreatitis using network pharmacology. Meanwhile, we conducted a meta-analysis from the perspective of evidence-based medicine to validate the efficacy of Dahuang Mudan Decoction in the treatment of acute pancreatitis.^[7,8]^

## 2. Materials and methods

### 2.1. Network pharmacology analysis

#### 2.1.1. Screening the active compounds

Drug potential active ingredient collection and target prediction: Dahuang Mudan Decoction contains 5 traditional Chinese medicines (Dahang, Mudan Pi, Taoren, Donggua Ren, and Mangxiao). Through the TCMSP (https://old.tcmsp-e.com/tcmsp.php) platform, oral bioavailability (OB) ≥ 0.3 and drug-like properties index (DL) ≥ 0.18 were used as the boundaries to screen the main active ingredients and targets of Dahuang Mudan Decoction. Based on the active ingredients and target genes of the TCMs screened from TCMSP, and supplemented accordingly by Swiss Target Prediction database (https://swisstargetprediction.ch/). At the same time, all the target genes queried in the database were organized and de-weighted.

#### 2.1.2. Establishment of a database between disease and targets

The target genes involved in the acute pancreatitis process were obtained from 2 databases: Gene Cards (https://www.genecards.org/), which contains a variety of biological information such as genomic, transcriptomic, proteomic, and genetic information. OMIM (https://www.omim.org/), Online catalogue of human genes and genetic diseases.

#### 2.1.3. Construction of the PPI network

The functional relationship between the ingredients in Dahuang Mudan Decoction and the targets involved in acute pancreatitis were visualized as a network using Cytoscape (version 3.7.1; https://www.cytoscape.org/). The nodes in the network represented the active constituents and the targets, and the edges represented the interactions between the active constituents and the targets.

Further, the interaction between these target proteins was shown as a network using an open-source online database, STRING (https://string-db.org/), which integrates a large number of known or predicted protein-protein interaction information in various organisms. Specifically, the ingredients in Dahuang Mudan Decoction and the targets involved in alopecia were uploaded to the STRING database (screened with confidence score > 0.4), and the study species were limited to“Homo sapiens.” Then the protein interaction relationships were downloaded as a TSV format, which could be visualized as the protein-protein interaction (PPI) network with Cytoscape. In the PPI network, the nodes represented proteins, and the edges represented the interactions.

#### 2.1.4. Bioinformatic annotation

The gene ontology (GO) and Kyoto Encyclopedia of Genes and Genomes (KEGG) were applied for the pathway enrichment analysis and the functional annotation analysis (https://metascape.org/gp/index.html). GO enrichment analysis included three categories: cellular component, biological process, and molecular function. Finally, the analytic results were represented with bar graphs, which were performed with an online bioinformatics tool (http://www.bioinformatics.com.cn/). In this study, *P* < .05 was considered statistically significant.

### 2.2. Meta-analysis

This study has been registered on PROSPERO. The registration number is CRD42024508770. Ethical approval is unnecessary because this is a literature-based study.

#### 2.2.1. Databases and search strategies

This study searched PubMed, Web of Science, Cochrane library, Wan Fang, VIP, and CNKI databases and the China Clinical Research Registry for clinical randomized controlled studies of previous applications of Dahuang Mudan Decoction for acute pancreatitis. Search terms included “Dahuang Mudan Decoction,” “Dahuang Mudanpitang,” “Dahuang Mudan Decoction Plus Reduction,” “Pancreatitis,” “AP,” “Severe Pancreatitis,” “Randomised Controlled Trial,” “Clinical Controlled Trial,” “Placebo,” “Clinical Trial,” “Controlled Trial,” and so on. Search formula: (“Dahuang Mudan Decoction” OR “Dahuang Mudanpi tang” OR “Dahuang Mudan Decoction Plus Reduction”) AND (“Pancreatitis” OR “acute pancreatitis” OR “Severe Pancreatitis”)

#### 2.2.2. Inclusion and exclusion criteria

**Literatures**: All papers were researched to the efficacy and safety of Dahuang Mudan Decoction in the treatment of acute pancreatitis with no language as well as time constraints. **Participants**: Patients with acute pancreatitis; denial of severe cardiac, pulmonary, hepatic, or renal insufficiency; allergy to Dahuang Mudan Decoction herbs and other herbal preparations; and no use of antibiotics or other medications for pancreatitis in the 2 weeks prior to treatment, and no comorbid serious infections. **Intervention**: There is no restriction on the mode of administration of Dahuang Mudan Decoction for acute pancreatitis, including oral intake and retention enema. There is no restriction on the therapeutic dose and duration of treatment. The control group includes conventional western medicine treatment or blood purification for acute pancreatitis, and the experimental group includes the addition of Dahuang Mudan Decoction or Dahuang Mudan Decoction plus reduction to the treatment plan of the control group. In addition, other symptom supportive treatments were not limited in both groups. **Exclusion criteria**: Case reports; animal studies; basic research; personal experience and review articles; incomplete data; duplication of literature; baseline conditions not assessed, etc.

#### 2.2.3. Primary outcome indicators and secondary indicators

Primary outcome indicators include overall treatment efficacy, secondary outcome indicators include length of hospital stay, improved levels of inflammatory factors.

#### 2.2.4. Risk of bias assessment

Two authors independently assessed the risk of bias as described in the Cochrane Handbook for the systematic review of interventions. We categorized potential bias as high, low or unclear. The following items were assessed: Random sequence generation, Allocation concealment, Blinding of participants and personnel, Blinding of outcome assessment, Incomplete outcome data, Selective reporting, other biases.

#### 2.2.5. Research selection and statistic collection

After excluding duplicate publications, both authors first screened articles based on title and abstract. Full-text versions of papers that meet the inclusion criteria are then retained, and data on patient characteristics, treatment details and clinical outcomes are extracted independently. Disagreements, if any, will be resolved by a third author.

#### 2.2.6. Statistical analysis and data synthesis

Review Manager 5.4 software provided by Cochrance Collaboration Network was used for meta-analysis. The counting data were expressed by Risk ratio (RR) and 95% confidence interval (95% CI). The continuous variable data were expressed by weighted mean difference (WMD) and 95% CI, and the test level was *P* < .05. *I*^2^ was used to analyze the heterogeneity of the included studies. If *I*^2^ ≤ 50%, *P* > .05, there was no heterogeneity, and fixed effect model was used. If *I*^2^ ≥ 50%, *P* < .05, If *I*^2^ ≥ 50%, *P* < .05, heterogeneity was considered and random effect model was used. Standard deviations and potential errors in labeling for certain indicators were calculated using methods provided by Cochrane.^[9]^ We further analyze the source of heterogeneity, if the heterogeneity is too obvious, descriptive analysis is used. Funnel plot was used to test potential publication bias.

## 3. Results

### 3.1. Screening of the active ingredients

According to the TCMSP database search, 44 active ingredients of traditional Chinese medicines were obtained with OB ≥ 0.30 and DL ≥ 0.18 as the critical values. The TCMSP search data and the active ingredient targets queried in Swiss Target Prediction database were combined and de-emphasized after standardized annotation in Uniprot database, eventually a total of 628 targets were obtained.

### 3.2. Acute pancreatitis target search and intersection target acquisition

**Figure 1.**
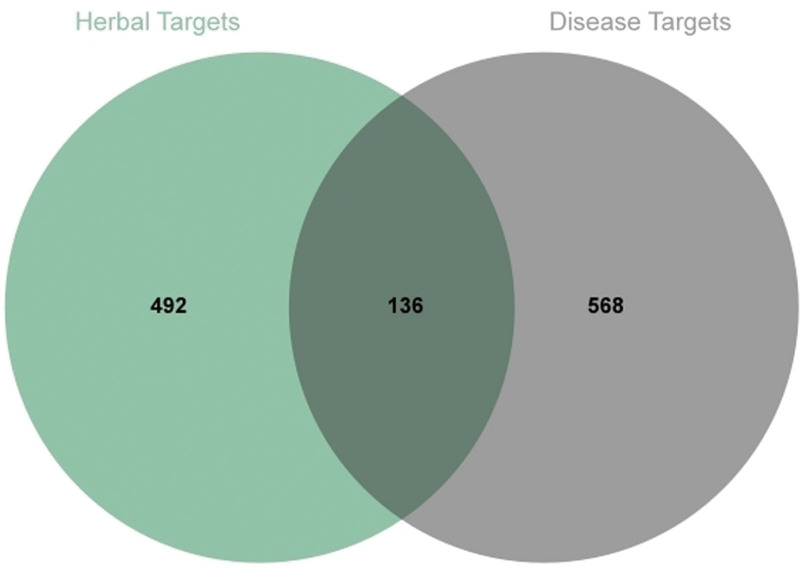
Venn diagram for screening of Chinese her ingredient targets and disease targets.

704 potential therapeutic targets were collected from OMIM, DrugBank, and GeneCards databases, which were intersected with the targets of traditional Chinese medicines, and 136 intersected targets were obtained by plotting the Venn diagram (Fig. 1).

### 3.3. TCM active ingredient-target network construction

**Figure 2.**
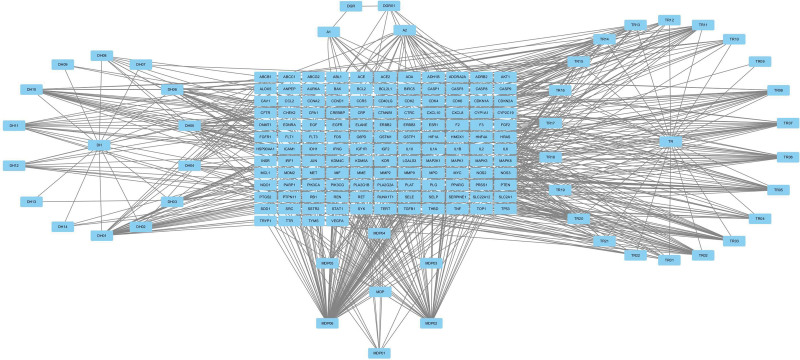
Construction of the compounds-target visual network of acute pancreatitis.

Using Cytoscape 3.7.2 software, we constructed a complex network diagram linking the components of traditional Chinese medicines with the intersection targets. Detailed compounds have been listed in order (Table 1). Statistically, the active ingredients with higher degrees include kaempferol, quercetin, and eupatin, which can be hypothesized to be key compounds primarily involved in the treatment of acute pancreatitis (Fig. 2).

**Table 1:** Chemical composition of Dahuang Mudan Decoction; DH01-DH14 represent the 14 active ingredients of Dahuang; MDP01-MDP06 represent the 6 active ingredients of Mudan Pi; TR01-TR22 represent the 22 active ingredients of Taoren; A1 and A2 represent the ingredients common to Dahuang Mudan Decoction.

Number	Ingredient name	Degree	Number	Ingredient name	Degree
DH01	Eupatin	30	TR03	2,3-didehydro Gallic acid 77	20
DH02	Mutatochrome	2	TR04	Gallic acid119	13
DH03	Physciondiglucoside	7	TR05	Gallic acid 120	24
DH04	Procyanidin B-5,3’-O-gallate	14	TR06	Gallic acid 121-isolactone	20
DH05	rhein	19	TR07	Gallic acid 122	19
DH06	Sennoside E_qt	23	TR08	Gallic acid 122-isolactone	20
DH07	Torachrysone-8-O-beta-D-(6’-oxayl)-glucoside	2	TR09	gibberellin 17	2
DH08	Toralactone	9	TR10	4a-formyl-7alpha-hydroxy-1-methyl-8-methylidene-4aalpha,4bbeta-gibbane-1alpha,10beta-dicarboxylic acid	9
DH09	Emodin-1-O-beta-D-glucopyranoside	3	TR11	Gallic acid 30	21
DH10	Sennoside D_qt	23	TR12	Gibberellin A44	11
DH11	palmidin A	14	TR13	Gallic acid 54	15
DH12	aloe-emodin	10	TR14	Gallic acid 60	13
DH13	gallic acid-3-O-(6’-O-galloyl)-glucoside	2	TR15	Gallic acid 63	17
DH14	(-)-catechin	4	TR16	gibberellin 7	18
MDP01	Mairin	9	TR17	Gallic acid 77	15
MDP02	kaempferol	47	TR18	Gallic acid 87	16
MDP03	(+)-catechin	3	TR19	3-O-p-coumaroylquinic acid	7
MDP04	benzoyl paeoniflorin	16	TR20	Populoside_qt	20
MDP05	5-[[5-(4-methoxyphenyl)-2-furyl]methylene]barbituric acid	9	TR21	hederagenin	14
MDP06	quercetin	85	TR22	campesterol	4
TR01	Sitosterol alpha1	10	DGR01	Stigmasterol	7
TR02	2,3-didehydro GA70	21	A2	beta-sitosterol	28
			A1	sitosterol	10

### 3.4. PPI network construction

**Figure 3.**
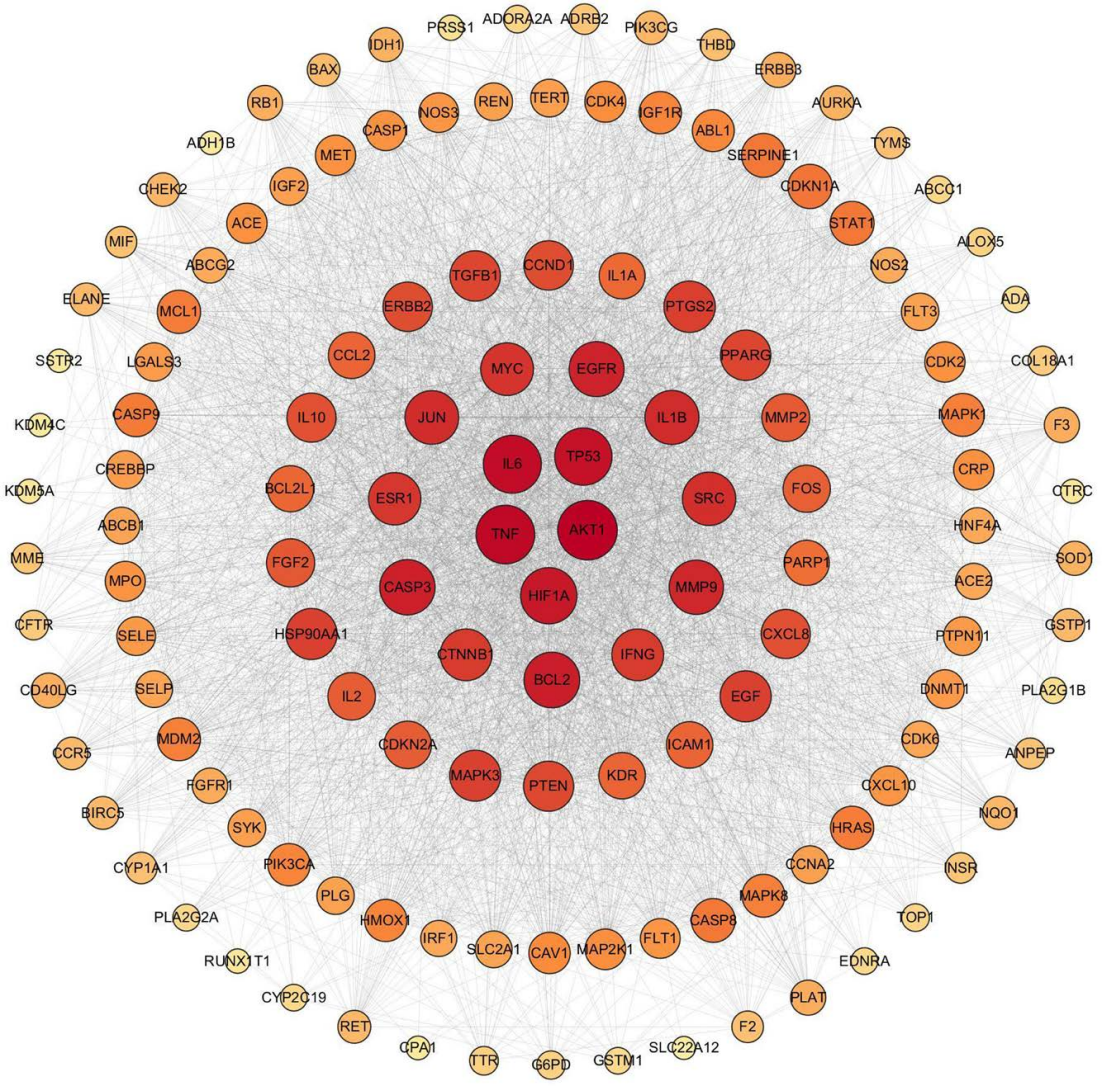
A diagram of the interactions between the active ingredient targets, where darker colors indicate that the targets have more complex interactions and may play a greater role in the treatment.

The intersecting targets were formed into a PPI network by STRING11.0 software (Fig. 3). Among them, AKT1, TNF, IL-6, TP53, HIF1A, BCL2, CASP3, EGFR, MMP9, IL1B has a more advanced degree ordering, so they are considered to be important targets on the protein interactions system (Table 2).

**Table 2:** The top 10 key targets of the degree value.

Gene name	Protein name	Degree
AKT1	AKT serine/threonine kinase 1	114
TNF	Tumor Necrosis Factor	111
IL6	Interleukin 6	109
TP53	Tumor protein p53	107
HIF1A	Hypoxia-inducible factor 1-alpha	104
BCL2	B-cell lymphoma-2	102
CASP3	Caspase 3	101
EGFR	Epidermal growth factor receptor	100
MMP9	Matrix metallopeptidase 9	98
IL1B	Interleukin-1 beta	97

### 3.5. Analysis of the biological functions and pathways of key target genes

**Figure 4.**
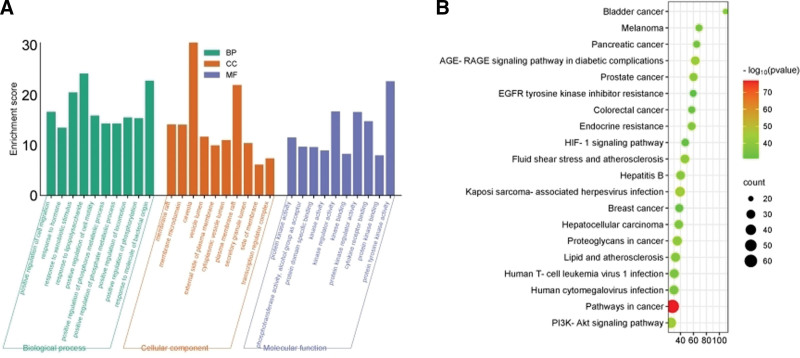
(A) GO enrichment analysis of cross genes between Dahuang Mudan Decoction and acute pancreatitis; (B) KEGG enrichment analysis of cross genes between Dahuang Mudan Decoction and acute pancreatitis. BP = biological process, CC = cell composition, GO = Gene ontology, KEGG = Kyoto encyclopedia of genes and genomes, MF = molecular function.

GO function enrichment analysis method was performed, yielding 1909 biological processes, 89 cellular components, and 172 molecular functions. Biological processes mainly include positive regulation of cell migration, response to hormones, and response to xenobiotic stimulus, among others. Cellular components mainly include membrane rafts, membrane microdomains and, and caveolae. Molecular functions mainly involve protein kinase activity, phosphotransferase activity, alcohol group as an acceptor, and protein domain specific binding etc (Fig. 4A). KEGG pathway enrichment analysis was conducted on the screened target proteins, resulting in a total of 195 enrichment outcomes. KEGG enrichment analysis of the key protein modules was performed to draw bubble diagrams of the top 20 pathways with the relevant pathway *P* values, in which the intersecting targets mainly acted on Pathways in cancer, Kaposi sarcoma-associated herpesvirus infection, PI3K-Akt signaling pathway and AGE-RAGE signaling pathway in diabetic complications, Fluid shear stress and atherosclerosis Proteoglycans in cancer (Fig. 4B).

### 3.6. Incorporation of literature statistics and basic information

**Figure 5.**
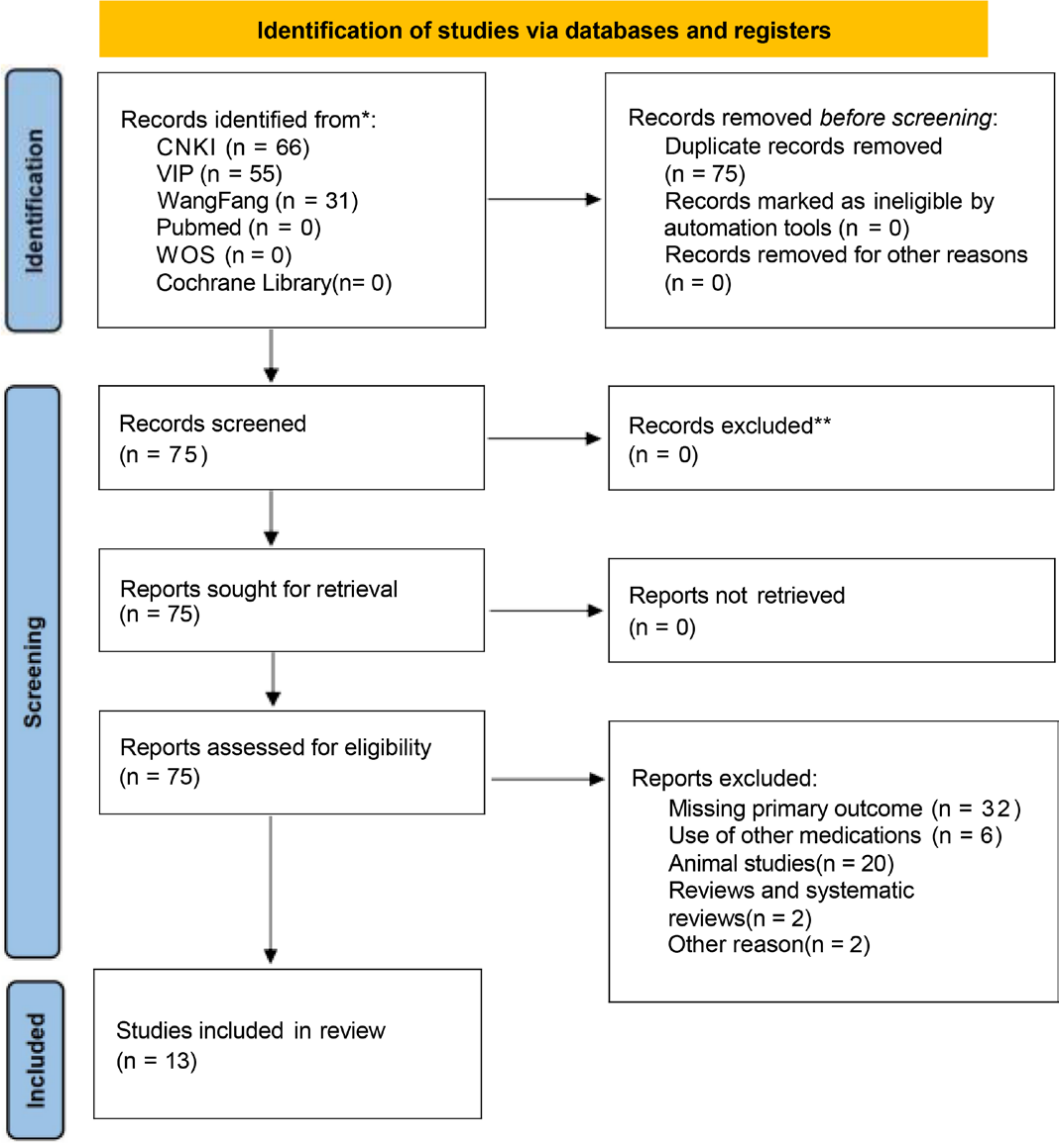
Study flow diagram for patient inclusion, screening, and final cohort selected for analysis.

**Figure 6.**
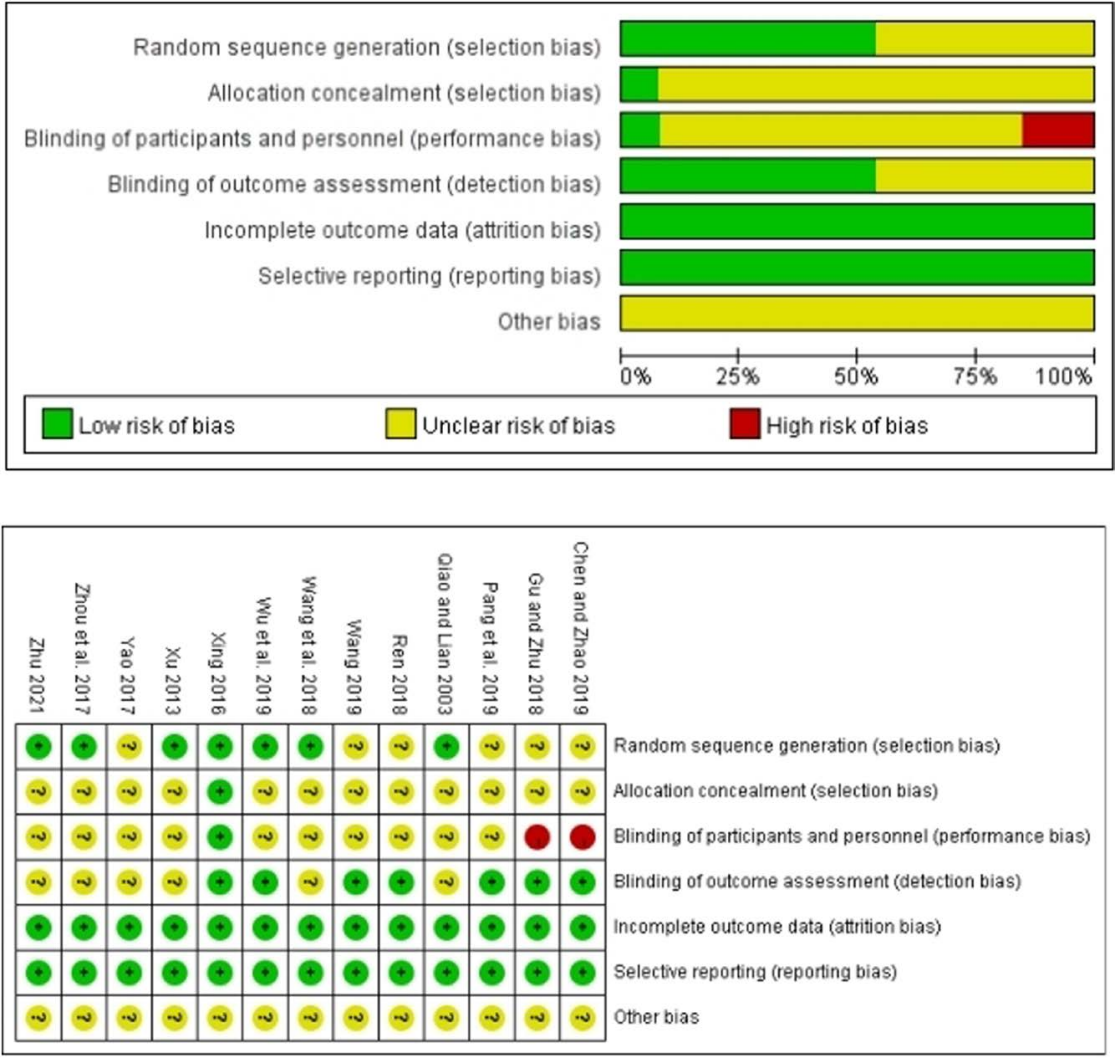
Cochrane collaboration risk of bias summary: evaluation of bias risk items for each included study.

As depicted in the flow chart of the meta-analysis, we conducted a comprehensive search strategy across 7 databases and the Chinese Clinical Research Registry. Initially, 152 relevant papers were identified. After excluding 2 reviews, 20 animal studies, 6 papers that did not align with the research objectives or diseases, and 32 papers lacking primary outcome data, a total of 13 randomized controlled trials were included in this study^[10–22]^ (Fig. 5). Baseline characteristics between the treatment and control groups were comparable across the trials. Eleven studies assessed overall treatment effectiveness, 8 studies evaluated improvement in inflammatory factors, and 4 studies measured hospitalization duration. Risk assessments for study inclusion were completed (Fig. 6). Basic information about the included studies was also summarized (Table 3).

**Table 3:** Basic information about the included studies.

Author/year	Number of case		Sex (Men/Women)		Age		EG	CG	Outcome indicator
	EG	CG	EG	CG	EG	CG			
Chen and Zhao 2019^[10]^	90	90	43/47	52/38	52.5 ± 6.9	52.5 ± 2.1	A + C	D	a + b
Gu and Zhu 2018^[11]^	29	29	18/11	21/8	54.27 ± 12.31	55.14 ± 10.72	A + F	F	b
Pang, et al 2019^[12]^	63	63	27/36	30/33	55.10 ± 3.10	55.30 ± 2.48	A + D	D	d
Qiao and Lian 2003^[13]^	35	23	14/21	14/11	48.5 ± 7.5	46.5 ± 9.5	A + D	D	b
Ren 2018^[14]^	40	40	29/11	28/12	54.34 ± 12.42	54.27 ± 12.38	B + D	D	a
Wang, et al 2018^[15]^	43	43	23/20	26/17	52.85 ± 11.40	50.27 ± 12.85	A + F + D	F + D	a + c + d
Wang 2019^[16]^	37	37	20/17	21/16	43.12 ± 1.12	43.15 ± 1.14	B + D	D	a + c + d
Wu, et al 2019^[17]^	29	29	18/11	17/12	50.7 ± 13.9	50.6 ± 13.8	B + D	D	a
Xing 2016^[18]^	50	50	26/24	31/19	36.38 ± 14.05	37.65 ± 13.14	A + D	D	a + c + d
Xu 2013^[19]^	30	30	18/12	19/11	22.10 ± 11.12	21.15 ± 12.35	A + D	D	a
Yao 2017^[20]^	40	40	21/19	20/20	36.4 ± 14.1	37.2 ± 14.0	A + F	F	a + b
Zhou, et al 2017^[21]^	65	65	39/26	40/25	48.65 ± 9.64	48.97 ± 8.67	B + D	D	a + c
Zhu 2021^[22]^	33	33	19/14	18/15	48.29 ± 5.64	48.91 ± 5.69	A + D	D	a + c

A: Chinese medicine retention enema; B: Oral or nasal administration of Chinese medicines; C: Somatostatin; D: Conventional treatment with western-style medicine; F: blood purification therapy; a: Overall response rate; b: Length of hospital stays; c: Serum TNF-a levels; d: Serum IL-6 levels.

CG = control group, EG = experimental group.

### 3.7. Overall response rate

**Figure 7.**
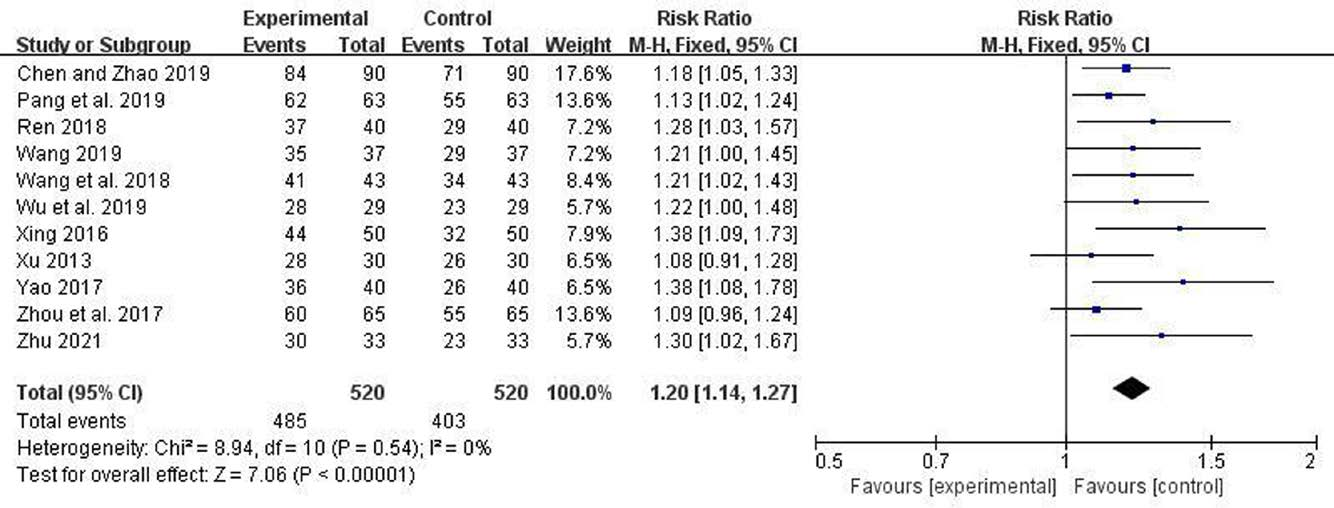
Forest plot comparing overall response rates between the 2 groups.

Eleven articles reported the overall response rate. There was no heterogeneity among the studies (*I*^2^ = 0%, *P* = .54), so a fixed-effect model was used. Meta-analysis indicated a statistically significant higher overall response rate in the trial group compared to the control group (RR = 1.20, 95% CI = 1.14–1.27, *P* < .00001) (Fig. 7).

### 3.8. Length of hospital stays and level of improvement in inflammatory factors

**Figure 8.**
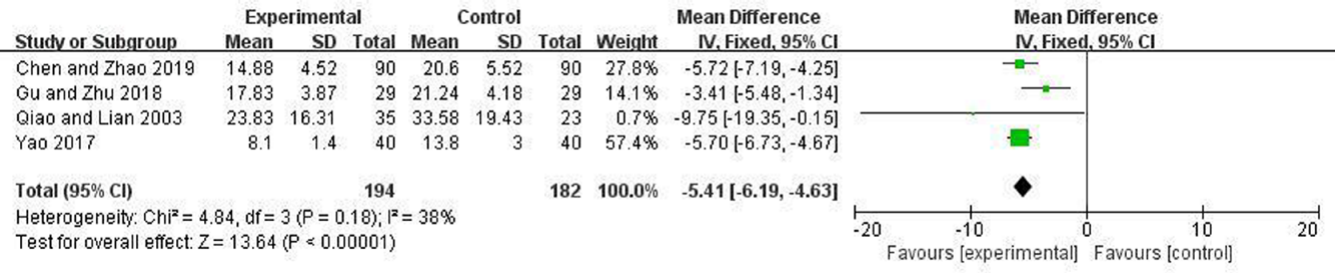
Forest plot comparing Length of hospitalization between the 2 groups.

**Figure 9.**
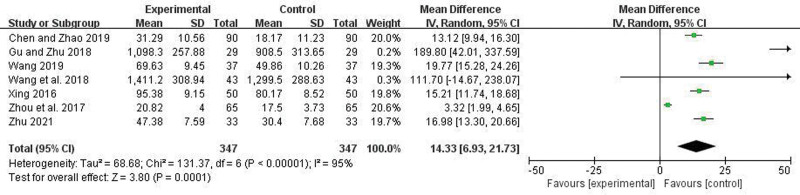
Forest plot comparing TNF-a between the 2 groups.

**Figure 10.**
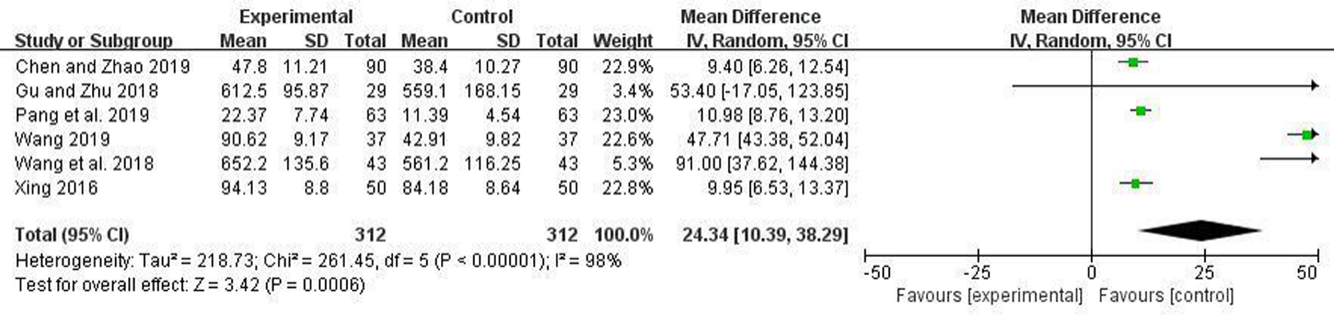
Forest plot comparing IL-6 between the 2 groups.

Four articles reported the Length of hospital stays. There was no heterogeneity among the studies (*I*^2^ = 38%, *P* = .18), so a fixed-effect model was applied. Meta-analysis showed that the hospitalization duration in the trial group was significantly less than in the control group, and the difference was statistically significant (OR = −5.41, 95% CI = −6.19 to −4.63, *P* < .00001) (Fig. 8). Seven articles reported the degree of improvement in TNF-a. There were heterogeneity among the studies (*I*^2^ = 95%, *P* < .00001), random effect model was used. Meta-analysis showed that the improved level of TNF-a in the trial group was more significant compared to the control group, and the difference was statistically significant (WMD = 14.33, 95% CI = 6.93–21.73, *P* = .0001) (Fig. 9). Six articles reported the degree of improvement in IL-6. There were heterogeneity among the studies (*I*^2^ = 98%, *P* < .00001), random effect model was used. Meta-analysis showed that the improved level of IL-6 in the trial group was more significant compared to the control group, and the difference was statistically significant (WMD = 24.34, 95% CI = 10.39–38.29, *P* = .0006) (Fig. 10).

### 3.9. Publication bias analysis

**Figure 11.**
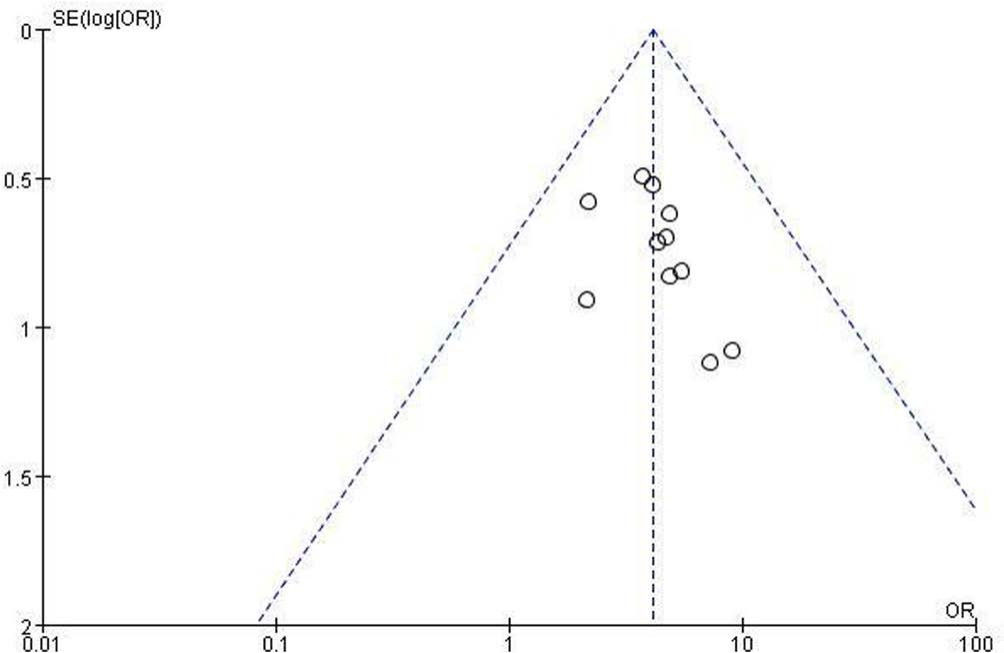
Funnel plot of all the included studies for assessing publication bias.

A funnel plot analysis was conducted to assess publication bias in the overall response rate. The plot exhibited good symmetry with scattered points evenly distributed within the funnel, indicating minimal influence of publication bias on the meta-analysis results (Fig. 11).

## 4. Discussion

As a common emergency in the emergency department, the effectiveness and timeliness of treatment for acute pancreatitis significantly influence patient prognosis. In particular, the risks associated with severe pancreatitis are currently receiving significant attention. There is a growing interest in the use of traditional herbal medicines and extracorporeal therapies. Studies have shown that Chinese herbal medicines have several advantages in the treatment of acute pancreatitis.^[23]^ Pancreatic injury is closely related to disturbances in intestinal function. A study on Chinese herbal recipe containing Dahuang showed that the use of Chinese herbs resulted in better recovery of intestinal function in patients with severe acute pancreatitis, as well as greater relief of their level of abdominal pain.^[24]^ Chinese herbal medicine restores the intestinal mucosal barrier and modulates protein kinase C-mediated calcium release from colonic smooth muscle in the intestines to regulate peristalsis and simultaneously clear intestinal toxins.^[25]^ Studies have shown that herbal enemas are also effective in the treatment of patients with acute pancreatitis.^[26]^ The treatment of acute pancreatitis with retained enemas of Dahuang Mudan Decoction is centered on activating blood, resolving stasis, clearing heat, and draining dampness, which is useful in the improvement of the disease.

In this study, preliminary screening of network pharmacology showed that the active ingredients of Dahuang Mudan Decoction for the treatment of acute pancreatitis were mainly kaempferol, quercetin and eupatin. Experimental studies showed that kaempferol could inhibit the activation of NF-kB signaling pathway to reduce the production of inflammatory factors associated with pancreatitis, attenuate the inflammatory response, and improve the prognosis.^[27]^ Meanwhile, kaempferol also inhibited the proliferation of human pancreatic cancer cells (PANC-1) and the protein expression levels of NF-kB and vascular endothelial growth factor (VEGF) in a dose-dependent manner, thereby inhibiting apoptosis and invasion of pancreatic cancer cells.^[28]^ Quercetin is a class of flavonoids with positive effects on the prevention of inflammation. It can regulate proteins involved in various oncogenic and signaling pathways. Additionally, it can inhibit tumor necrosis factor-a (TNF-a) and the p38/MAPK signaling pathway through the up-regulation of miR-216b, thus having a protective effect on acute pancreatitis.^[29]^ Moreover, quercetin can inhibit the migration activity of pancreatic cancer cells induced by epidermal growth factor.^[30]^ Eupatin also has significant anti-inflammatory and neuroprotective effects.^[31]^

The main feature of pancreatitis is that pancreatic enzymes cause pancreatic auto-digestion, which leads to autophagy and apoptosis of pancreatic acinar cells. These effects may trigger a severe inflammatory response and release inflammatory factors. Inflammatory cells and pancreatic acinar cells then release a variety of inflammatory mediators, including pro-inflammatory cytokines such as interleukin-6 (IL-6) and tumor necrosis factor-alpha (TNF-a).^[32]^The increase in the level of these factors leads to an increase in vascular permeability, activation of leukocytes, and destruction of tissues. The release of inflammatory mediators is the key factor in the pathogenesis of pancreatitis, and it can aggravate the condition, leading to the involvement of organs outside the pancreas.^[33]^

Relevant studies have provided strong evidence for clinical disease diagnosis through the screening or modeling of target genes, indicating the importance of target genes in disease development.^[34]^ The top 10 targets according to network pharmacology were AKT1, TNF-a, IL-6, TP53, HIF1A, BCL2, CASP3, EGFR, MMP9, IL1B. Among them, IL-6 and TNF-a are important regulators in the inflammatory response.^[35]^ On the one hand, IL-6 can regulate the inflammatory response by phosphorylating STAT3 at specific sites through the MAPKAPK-2 signal transduction pathway, allowing it to enter the nucleus and bind to chromosomal DNA.^[36]^ On the other hand, TNF-a is an important pro-inflammatory factor. A rapid rise in its level will lead to the aggravation of pancreatitis, and inhibition of it will cause apoptosis of pancreatic acinar cells, resulting in a significant reduction of pancreatitis.^[37]^

Aggregations of different cells may have different immune landscapes or KEGG expression.^[38]^ KEGG enrichment analysis of the key protein module of the relevant pathways ranked in the top 20 by *P* value was visualized using a bubble diagram, in which the intersection of the targets are mainly in the AGE-RAGE signaling pathway in diabetic complications, the PI3K-Akt signaling pathway, etc.

Diabetic complications of the AGE-RAGE signaling pathway can activate NF-kB to cause the expression and release of a large number of adhesion molecules, growth factors, and pro-inflammatory factors. This can lead to inflammation and oxidative stress. Therefore, the main components of traditional Chinese medicine act on this signaling to a certain extent, influencing the development of acute pancreatitis.^[39]^ The PI3K-Akt signaling pathway can regulate apoptosis, oxidative stress, autophagy, and more. Wang et al^[40]^ found that the activation of PI3K signaling pathway could be inhibited by propargylglycine in severe acute pancreatitis (SAP) rats. This inhibition reduced the activity of NF-kB, which led to the inhibition of apoptotic genes, a reduction in the release of inflammatory factors, and alleviation of the clinical symptoms of acute pancreatitis. Laura et al^[41]^ showed that PI3K/Akt signaling pathway can activate NF-kB to induce oxidative stress in alveolar cells, and at the same time mediate the downstream pathway leading to inflammatory reactions in the pancreas. Another study showed that rhubarbin contained in the traditional Chinese medicine Dahuang can inhibit autophagy-related genes and reduce the severity of acute pancreatitis in rats.^[42]^ All of the above can illustrate the important role of the main signaling pathways in acute pancreatitis, and the results of network pharmacology show that they can be influenced by the main active ingredients of traditional Chinese medicine.

The results of the meta-analysis showed that the treatment group had a significant advantage in terms of overall efficacy, level of improvement in inflammatory factors, and shorter hospital stays compared with the control group. The effect of Dahuang Mudan Decoction on various of inflammatory factors can be further supported.

However, there were some shortcomings in this study, including the lack of mention of blinding and allocation concealment in the 13 included studies. In addition, the methodological quality of the included studies was average, and gray literature such as unpublished studies was not included. The overall sample size for the systematic evaluation was small, and assessments of adverse effects and safety were lacking. The limitations of the Network Pharmacology TCMIP platform database may have resulted in an incomplete collection of active ingredients of drugs. Therefore, future studies need to expand the sample size and optimize the clinical trial specifications to better leverage the advantages of traditional Chinese medicine in clinical practice. Finally, further verification of the specific mechanisms behind the results of this study is necessary through in vivo and in vitro experiments.

## 5. Conclusion

In summary, network pharmacological studies have shown that Dahuang Mudan Decoction affects acute pancreatitis through multiple components, targets and mechanisms. In addition, evidence-based medicine provides strong support for the effectiveness of Dahuang Mudan Decoction in the treatment of acute pancreatitis.

## Author contributions

**Conceptualization:** Jinhan Chen, Mengjie Jiang, Mingxian Chen.

**Data curation:** Jinhan Chen, Mengjie Jiang, Yuou Ying, Yuan Ji, Yuying Chi, Linghui Tao, Fuping Wu.

**Investigation:** Yuan Ji, Yuying Chi, Linghui Tao, Fuping Wu.

**Methodology:** Jinhan Chen, Mengjie Jiang, Yuou Ying, Mingxian Chen.

**Supervision:** Jinhan Chen.

**Writing – original draft:** Jinhan Chen, Mengjie Jiang, Mingxian Chen.

**Writing – review & editing:** Jinhan Chen, Mingxian Chen.
